# Drug-induced subacute cutaneous lupus erythematosus secondary to Dupilumab: A case report

**DOI:** 10.1177/2050313X241284049

**Published:** 2024-09-25

**Authors:** Miranda Waugh, Geneviève Gavigan

**Affiliations:** The Division of Dermatology, The University of Ottawa, Ottawa, ON, Canada

**Keywords:** Dupilumab, subacute cutaneous lupus erythematosus, drug induced

## Abstract

We present the case of a 63-year-old male with known rheumatoid arthritis, diabetes, hypertension, and peripheral neuropathy who developed drug-induced subacute cutaneous lupus erythematosus secondary to Dupilumab for his chronic hand dermatitis.

## Introduction

Dupilumab is a widely used monoclonal antibody medication used for the treatment of atopic dermatitis, asthma, eosinophilic esophagitis, prurigo nodularis, and rhinosinusitis with nasal polyps, by inhibiting interleukin (IL)-4 and IL-13 in the Th2 pathway.^1^ For this reason, it may be used in the treatment of refractory chronic hand dermatitis when an underlying atopic diathesis is driving disease activity. Although it has a favorable safety profile, the literature brings to light many cutaneous adverse events linked to this medication including facial erythema, paradoxical psoriasis, alopecia, drug hypersensitivity reactions, and cutaneous T-cell lymphoma.^2^ There are currently many immunological hypotheses explaining the development of these conditions through compensatory mechanisms such as shifting of the Th2 pathway to the Th1 pathway and Th2 and Th17 imbalance, which both have been mentioned as possible causes for the development of discoid lupus erythematosus and systemic lupus erythematosus secondary to Dupilumab.^2^

Despite the fact it is poorly understood, drug-induced subacute cutaneous lupus has been frequently reported with numerous well-known medications in the antihypertensive, antifungal, proton pump inhibitor, and chemotherapy drug classes.^3^ Less commonly, biologics have been associated with the development of drug-induced subacute cutaneous lupus erythematosus (DI-SCLE) however mostly with anti-tumor necrosis factor alpha medications.^3^ A photodistributed papulosquamous or annular eruption in addition to positive antinuclear antibodies are both important diagnostic features of DI-SCLE; however, there are no formal diagnostic criteria for this entity.^3^ In current literature, Dupilumab has only once been associated with the development of drug-induced subacute cutaneous lupus erythematosus, confirmed histologically, clinically, and by complete resolution of symptoms after drug cessation.^4^ We present the second case of a patient who presents with drug-induced subacute cutaneous lupus erythematosus secondary to Dupilumab that resolved post-drug cessation.

## Case report

The patient of interest was referred to Dermatology after his chronic hand dermatitis was failing to respond to regular application of Clobetasol ointment twice a day. His main symptoms were pain secondary to fissures and extensive scaling. He was transitioned to topical Crisaborole 2% ointment twice a day and topical Tacrolimus 0.1% ointment twice a day afterward as maintenance with only partial improvement. For this reason, treatment was escalated to systemic weekly Methotrexate in combination with topical Desoximetasone cream twice a day to the affected areas. On Methotrexate, he experienced partial relief however continued to have significant flares of his disease. Subsequently, Dupilumab was introduced, leading to an initial improvement in his symptoms. However, approximately 4 months into his course of Dupilumab, he began to develop intraoral pain and a new skin eruption affecting primarily his trunk. The eruption consisted of multiple urticoid erythematous papules admixed with ill-defined red scaly annular plaques in a photodistributed pattern. A punch biopsy was performed and revealed a superficial perivascular lymphocytic inflammation with interface dermatitis. The blood work of relevance revealed an antinuclear antibody of 1:160 speckled pattern, a negative ds-DNA, and weakly positive anti-histone antibodies consistent with drug-induced SCLE. The decision to discontinue Dupilumab resulted in a resolution in his truncal eruption, but persistent dry mouth and tongue discomfort prompted a referral to rheumatology for further evaluation of his symptoms.

## Discussion

This case outlines a significant and rare adverse event secondary to the start of Dupilumab for chronic hand dermatitis. As per current literature, this appears to be the second reported case of DI-SCLE to Dupilumab for dermatitis.^4^ The diagnosis was confirmed by his clinical presentation, positive ANA, and anti-histone antibodies together with a skin biopsy. More convincing that his skin eruption and intraoral pain resolved after drug cessation. Of note, his anti-Ro/SS-A and anti-La/SS-B antibodies were negative, two antibodies that are usually correlated with DI-SCLE; however, his positive anti-histone antibodies remain a serologic pertinent positive toward the diagnosis.^3^ It is important to mention the patient was not autoimmune disease naïve which could have possibly played a role in his presentation. Moving forward, it will be important to monitor him for symptom re-occurrence despite avoiding Dupilumab. More cases and research are needed to further understand the immunologic pathways leading to the development of various forms of lupus secondary to Dupilumab.
